# Case Report: Acute Brucellosis Due to *Brucella suis* in a Brazilian Gold Miner Diagnosed in French Guiana

**DOI:** 10.4269/ajtmh.22-0228

**Published:** 2023-05-30

**Authors:** Alessia Melzani, Mathilde Boutrou, Vincent Sainte-Rose, Frédégonde About, Maylis Douine, Céline Michaud, Mathieu Nacher, Mélanie Gaillet, Denis Blanchet, Jean Philippe Lavigne, Magalie Demar, David O’Callaghan, Felix Djossou, Anne Keriel, Loïc Epelboin

**Affiliations:** ^1^ Unité de Maladies Infectieuses et Tropicales, Centre Hospitalier de Cayenne, Cayenne, French Guiana;; ^2^ Laboratoire Hospitalo-Universitaire de Microbiologie, Centre Hospitalier de Cayenne, Cayenne, French Guiana;; ^3^ Centre d’Investigation Clinique Antilles-Guyane, INSERM CIC 1424, Cayenne, French Guiana;; ^4^ Centres Délocalisés de Prévention et de Soins, Centre Hospitalier de Cayenne, Cayenne, French Guiana;; ^5^ Virulence Bactérienne et Infections Chroniques (VBIC), U1047, INSERM, Université de Montpellier, Nîmes, France;; ^6^ Centre National de Référence des Brucella, Service de Microbiologie, Centre Hospitalier Universitaire de Nîmes, Nîmes, France

## Abstract

A 29-year-old Brazilian illegal gold miner developed intermittent fever. Blood cultures were positive for Gram-negative coccobacilli and, after an initial misidentification by an automated identification system, the diagnosis of brucellosis caused by *Brucella suis* was confirmed. We hypothesize an association with domestic or wild swine exposure. The patient responded well to standard antibiotic therapy of brucellosis. We report the first case of human brucellosis on the Guiana Shield. This report underlines the importance of considering brucellosis in the presence of a fever of unknown origin, even in the Amazonian rainforest area, where several zoonotic diseases might be considered in the differential diagnosis of unexplained fever.

## INTRODUCTION

Brucellosis is a neglected global zoonosis caused by bacteria of the genus *Brucella*. Four main species of *Brucella* are considered to cause human brucellosis: *Brucella abortus* (cows), *Brucella melitensis* (goats and sheep), *Brucella suis* (swine), and *Brucella canis* (dogs). People become infected through direct contact with infected animals, consumption of unpasteurized dairy products or undercooked meat, or inhalation of contaminated aerosols.^1^

Human brucellosis can present with acute, subacute, and chronic forms; initial symptoms can include fever, weakness, arthromyalgia, and anorexia. Some patients can develop localized complications (endocarditis, arthritis, spondylitis, neurological manifestations, etc.), and symptoms depend on the site of involvement.^1^^,^^2^

Human brucellosis is traditionally considered endemic in South America, particularly in Peru and Argentina,^3^^,^^4^ and is becoming a public health concern in the State of Paraná, in Brazil.^5^ Few South American data on this pathology have been published.^6^ Although a study conducted in slaughterhouses of Venezuela in 2006 found a seroprevalence of *Brucella* antibodies of 5.6% and 11.2% among 159 workers and 303 cattle, respectively,^7^ to our knowledge no cases of human brucellosis have been reported on the Guiana Shield. The Guiana Shield is a geological region located on the northeastern coast of the South American continent, which includes, from west to the east, the eastern Venezuelan State of Bolivar, Guiana (formerly British Guiana), Suriname (formerly Dutch Guiana), French Guiana, the Brazilian State of Amapá, and a part of the State of Pará. French Guiana is a French overseas region where socioeconomic and health indicators are higher than in neighboring countries. This article aims to describe the first human case of brucellosis, diagnosed in French Guiana in 2017.

## CASE REPORT

A 29-year-old Brazilian man presented to the health center of Maripasoula (a town along the Maroni River, which separates French Guiana from Surinam, and located 300 km away from Cayenne, the main city of French Guiana) in August 2017 with fever, headache, chest, abdominal, and lower back pain, diarrhea, and loss of 2 kg within a few days. He had arrived 1 month prior from Santa Helena, a municipality of the State of Maranhão (northeastern Brazil), to work in an illegal gold mine in the Amazonian forest of French Guiana. He had no medical history. The patient used to have animals at home in Brazil, including pigs. He also declared consuming hunted meat. He was afebrile. Clinical examination revealed no abnormality except inguinal lymph node enlargement. Blood samples for routine tests and cultures were taken and the patient was discharged.

Laboratory tests showed the following: white blood cell (WBC) count 2.2 giga (G)/L, neutrophils 1.04 G/L, lymphocytes 0.76 G/L, hemoglobin 12.9 g/dL, mean corpuscular volume 84 µm^3^, platelets 127 G/L, C-reactive protein (CRP) 53.4 mg/L, aspartate transaminase 74 IU/L (*N* < 41 IU/L), and alanine transaminase 71 IU/L (*N* < 40 IU/L). HIV serology was positive with a viral load of 7,115 copies/mL (3.85 log) and CD4 lymphocytes 0.315 G/L (46.1%). Blood cultures were positive with Gram-negative rods after 6 days of incubation. Matrix-assisted laser desorption/ionization time-of-flight mass spectrometry (MALDI-TOF MS) (Biotyper, Bruker Daltonics, Billerica, MA) did not allow identification, but the VITEK^®^ 2 GN ID card identified colonies as *Ochrobactrum anthropi* with 99% probability. Upon reception of the results, on day 7 after the first examination the patient was invited to consultation again at the health center. He was asymptomatic. A blood sample was taken and the patient went back home. The second blood culture was again positive for *O. anthropi*. Despite the results of the blood culture, it was not possible to transport the patient to Cayenne Hospital, because it was not possible to contact him until he returned to the health center on day 22 after the first admission.

He was admitted to Cayenne Hospital on day 23. He was asymptomatic and the blood tests were almost normalized. Two new blood cultures were carried out and were negative, and consequently no specific treatment was initiated; considering his good clinical condition and the progressive normalization of CRP level, a simple monitoring program was decided upon.

On day 41, the patient showed signs of sepsis: fever, lower back pain, and inflammation, with CRP at 150.9 mg/L, WBC count 1.1 G/L, neutrophils 0.5 G/L, lymphocytes 0.51 G/L, hemoglobin 10.4 g/dL, and platelet count 91 G/L. An antibiotic therapy based on an antimicrobial sensitivity test of the previous cultures was started with intravenous piperacillin-tazobactam (4 g, three times a day) and amikacin (15 mg/kg, once a day). Blood cultures were again identified as *O. anthropi*. A total of seven sets of blood cultures were positive.

Chest-abdomen-pelvis computed tomography was normal, lumbar spine magnetic resonance imaging scanning was negative for spondylodiscitis, and transesophagic echocardiography was negative for native valve endocarditis.

In view of the overall clinical presentation and the low probability of a nosocomial infection, the diagnosis of brucellosis began to be considered. Brucellosis agglutination tests were then performed and found to be positive. MALDI-TOF MS spectra were also reanalyzed using Bruker’s security-relevant library, which allows the identification of several highly pathogenic microorganisms. This identified *B. melitensis* with a good score (+2.18), thus confirming the diagnosis of brucellosis. The antibiotic was thus switched 12 days later to oral rifampicin (900 mg/day) and doxycycline (100 mg, twice daily) for 6 weeks. The patient recovered and was discharged. He continued antibiotics at home with regular follow-up at Maripasoula Health Center for HIV infection.

The bacterial isolate was sent to the *Brucella* National Reference Centre (CNR *Brucella*) for confirmation of identification. Reanalysis by MALDI-TOF MS using a VITEK mass spectrometer (bioMérieux, Marcy-l’Étoile, France) with the up-to-date in vitro diagnostic database v. 3.2.0^8^ confirmed that the isolate belonged to the *Brucella* genus (score 99.9%). This isolate formed nonhemolytic, round, convex, smooth, grayish colonies with a diameter of approximately 0.5 mm after 48  hours of incubation on tryptic soy agar plates (bioMérieux, Marcy-l’Étoile, France). Bacteria showed positive reactions for catalase and cytochrome oxidase and negative results for indole production. Multiplex typing polymerase chain reaction (PCR) was finally performed on the genomic DNA of this isolate, which was named BRSO-2017-022. The patterns obtained using Bruce-ladder PCR and Suis-ladder PCR^9^ showed that this isolate is in fact *B. suis* biovar 1.

## DISCUSSION

This is the first case of human brucellosis ever reported from the Guiana Shield. The patient was a Brazilian illegal gold miner who had been working in French Guiana for 1 month in the deep rainforest. The incubation period of brucellosis is usually between 2 and 4 weeks,^10^ but can be very long (several weeks to months). Bacteremia may occur at the early stage of the disease but also late in case of complications. This patient presented with an undulant fever with intermittent bacteremia and without localized complications, evolving in less than 2 months, compatible with an acute or subacute stage.

Because *B. suis* biovar 1 was the species identified, contact with swine or wild boars or ingestion of food products from these animals should be primarily considered. In the present case, this strain was probably acquired from pigs at the patient’s house in the Brazilian State of Maranhão, although it cannot be excluded that he contracted it upon contact with wild boars hunted in the Amazonian rainforest of French Guiana in the previous month. However, the host specificity of *Brucella* species is relative, and other animal sources should also be considered.

Brucellosis can be difficult to diagnose for several reasons. Even in countries where this zoonosis is endemic, other diseases such as malaria, typhoid, leptospirosis, and Q fever are often the first to come to mind. French Guiana has the highest incidence of acute Q fever, caused by *Coxiella burneti*, in the world,^11^^,^^12^ and patients with febrile illness are often empirically treated with doxycycline. Although *Brucella* are sensitive to doxycycline, it should not be used as a monotherapy for this infection. *Brucella* spp. are also slow-growing bacteria, and blood cultures can require up to 14 days to turn positive. Laboratory misidentification is also a problem; although clinical MALDI-TOF MS databases now correctly identify *Brucella*,^8^ routine tools such as VITEK^®^ 2 regularly misidentify these bacteria as the closely related *Ochrobactrum* sp.^13^^–^^15^ This is further complicated by the recent reclassification of *Ochrobactrum*, *Pseudochrobactrum*, and *Falsochrobactrum* as *Brucella*.^16^ Therefore, identification of *Ochrobactrum* in a clinical context evoking brucellosis should lead to serological tests for brucellosis and molecular analysis for confirmation of identification. In the present case, the misidentification of the species has two main explanations: first, brucellosis has never been identified before in our region, and second, at this moment there was no qualified bacteriologist at the hospital, with interpretation of the routine tools relying on laboratory technicians and biologists from other specialties (parasitology and mycology).

The isolation of *Brucella* and the antibiogram carried out before the identification of the bacterium represent a real risk of laboratory-acquired brucellosis for the personnel handling these cultures. If *Brucella* had been identified from the beginning, an antibiogram would not have been necessary because of the rarity of acquired resistance and the risk of transmission.

## CONCLUSION

This is the first case of human brucellosis reported from the Guiana Shield. Brucellosis can be misdiagnosed due to confusion with other common causes of fever in its acute presentation, or with tuberculosis in its subacute form. *Brucella* should henceforth be one of the infections to be considered when encountering acute fever in this Amazonian region.
